# Infectious diseases, infection control, vaccines and long-term care: an European interdisciplinary Council on ageing consensus document

**DOI:** 10.1007/s40520-025-03271-6

**Published:** 2025-12-22

**Authors:** Nicola Veronese, Jane Barratt, Eveline Coemans, Pete Dayananda, Marco Del Riccio, Tamas Fulop, Giovanni Gabutti, Stefan Gravenstein, Mickael Hiligsmann, Eva Hummers, George Kassianos, Francesco Macchia, Paolo Manzoni, Finbarr C. Martin, Jean-Pierre Michel, Alessandro Morandi, Jerome Ory, Jade Pattyn, Eva Peetermans, Maria Cristina Polidori, Matteo Riccò, Cornel Christian Sieber, Antoni Torres, Gerrit Adrianus van Essen, Stefania Maggi

**Affiliations:** 1 Department of Medicine, Geriatrics Section, University of Palermo, Palermo, Italy; 2 Faculty of Medicine, Saint Camillus University, Rome, Italy; 3 Unità Locale Socio 3 Serenissima, Primary Care Department, Venice, Italy; 4 Toronto, Canada; 5 Faculty of Medicine and Life Sciences, Hasselt University, Agoralaan, Diepenbeek, 3590 Belgium; 6 Department of Infection Prevention and Control & Hospital Outbreak Support Team, Jessa Hospital, Stadsomvaart 11, Hasselt, 3500 Belgium; 7 Department of Infectious Disease, Imperial College London, London, UK; 8 Department of Health Sciences, University of Florence, Florence, 50134 Italy; 9 Adult Immunization Board, Antwerp, Belgium; 10 Italian Scientific Society of Hygiene, Preventive Medicine and Public Health (SItI), Cogorno (Ge), Italy; 11 Division of Nephrology, Department of Medicine, Medical University of South Carolina, Charleston, SC USA; 12 Board of the Italian Vaccination Calendar for Life, Cogorno (Ge), Italy; 13 Alpert Medical School, School of Public Health, Brown University, Providence, USA; 14 Department of Health Services Research, CAPHRI Care and Public Health Research Institute, Maastricht University, Maastricht, The Netherlands; 15 Department of General Practice, University Medical Center Göttingen, Göttingen, Germany; 16 National Immunisation Lead Royal College of General Practitioners, British Global and Travel Health Association, Bath, UK; 17 HappyAgeing, Ancona, Italy; 18 SCDU Pediatria e Neonatologia, Ospedale Degli Infermi, Dipartimento Materno-Infantile ASLBI, Ponderano, 13875 BI Italy; 19 School of Medicine, University of Turin, Turin, 10124 TO Italy; 20 Population Health Sciences, Faculty of Life Sciences and Medicine, King’s College London, London, SE1 9RT UK; 21 Centre Medical, University of Geneva, 40A route de Malagnou, Genève, 1208 Switzerland; 22 Deparment of Clinical and Experimental Science, University of Brescia, Brescia, Italy; 23 Intermediate Care, Azienda Speciale Cremona Solidale, Cremona, Italy; 24 Group Medical Department, EMEIS, Puteaux, France; 25 Centre for the Evaluation of Vaccination, Vaccine & Infectious Disease Institute, University of Antwerp, Drie Eikenstraat 663, Edegem, 2650 Belgium; 26 Clinical Trial Center, Vesalius Hospital, Hazelereik 51, Tongeren, 3700 Belgium; 27 Aging Clinical Research, Department II of Internal Medicine and Center for Molecular Medicine Cologne, Faculty of Medicine, University of Cologne, University Hospital Cologne, Cologne, Germany; 28 Cologne Excellence Cluster on Cellular Stress Responses in Ageing- Associated Diseases (CECAD), University of Cologne, Cologne, Germany; 29 Servizio di Prevenzione e Sicurezza Negli Ambienti di Lavoro (SPSAL), AUSL-IRCCS di Reggio Emilia, Via Amendola 2, Reggio Emilia, 42122 Italy; 30 Institute for Biomedicine of Aging (IBA), Friedrich-Alexander-Universität Erlangen-Nürnberg, Erlangen, Germany; 31 CMO, County Hospital Winterthur, Winterthur, Switzerland; 32 Universitat de Barcelona, IDIBAPS, CIBERES, Barcelona, Spain; 33 The Dutch Immunisation Foundation, Amersfoort, The Netherlands; 34 National Research Council, Institute of Neuroscience, Padua, Italy

**Keywords:** Long-Term care, Aged, Vaccination, Communicable disease control, Immunization programs

## Abstract

The accelerating ageing of populations worldwide presents profound challenges for public health, particularly within long-term care facilities (LTCFs). Older adults, often burdened by multimorbidity, frailty, and immunosenescence, are highly vulnerable to vaccine-preventable diseases such as influenza, pneumococcal pneumonia, COVID-19, respiratory syncytial virus (RSV), pertussis, and herpes zoster (HZ). Despite the availability of effective vaccines, immunization coverage in LTCFs remains inadequate, hindered by fragmented national policies, insufficient mandates, and systemic neglect of adult vaccination. In many settings, vaccination uptake is not even systematically monitored, leaving policymakers and clinicians without reliable data to identify gaps or measure progress. The COVID-19 pandemic underscored these vulnerabilities, temporarily spurring emergency vaccination efforts but failing to establish sustainable, life-course immunization frameworks. This consensus document, developed by the European Interdisciplinary Council on Ageing (EICA) following the San Servolo (Venice, Italy) 2025 meeting, synthesizes evidence on intrinsic and environmental infection risk factors in LTCFs, the health and economic burden of infections, and the persistent gaps in vaccine uptake among both residents and staff. We highlight the cost-effectiveness of preventive interventions, the critical role of non-pharmacological infection control measures, and the need to address antimicrobial resistance through integrated vaccination strategies. The Council emphasizes that routine adult vaccination must become a structural element of care planning for ageing populations, supported by digital registries, systematic assessments at LTC admission, co-administration strategies, and robust staff engagement. Stronger global and national policy leadership is urgently needed to align LTCF immunization with life-course approaches and primary healthcare integration. Protecting frail older adults from infectious diseases is not only a clinical necessity but also a societal obligation—central to safeguarding dignity, resilience, and healthy ageing in Europe and beyond.

**Graphical Abstract:**

The fi gure was made FigureLabs.
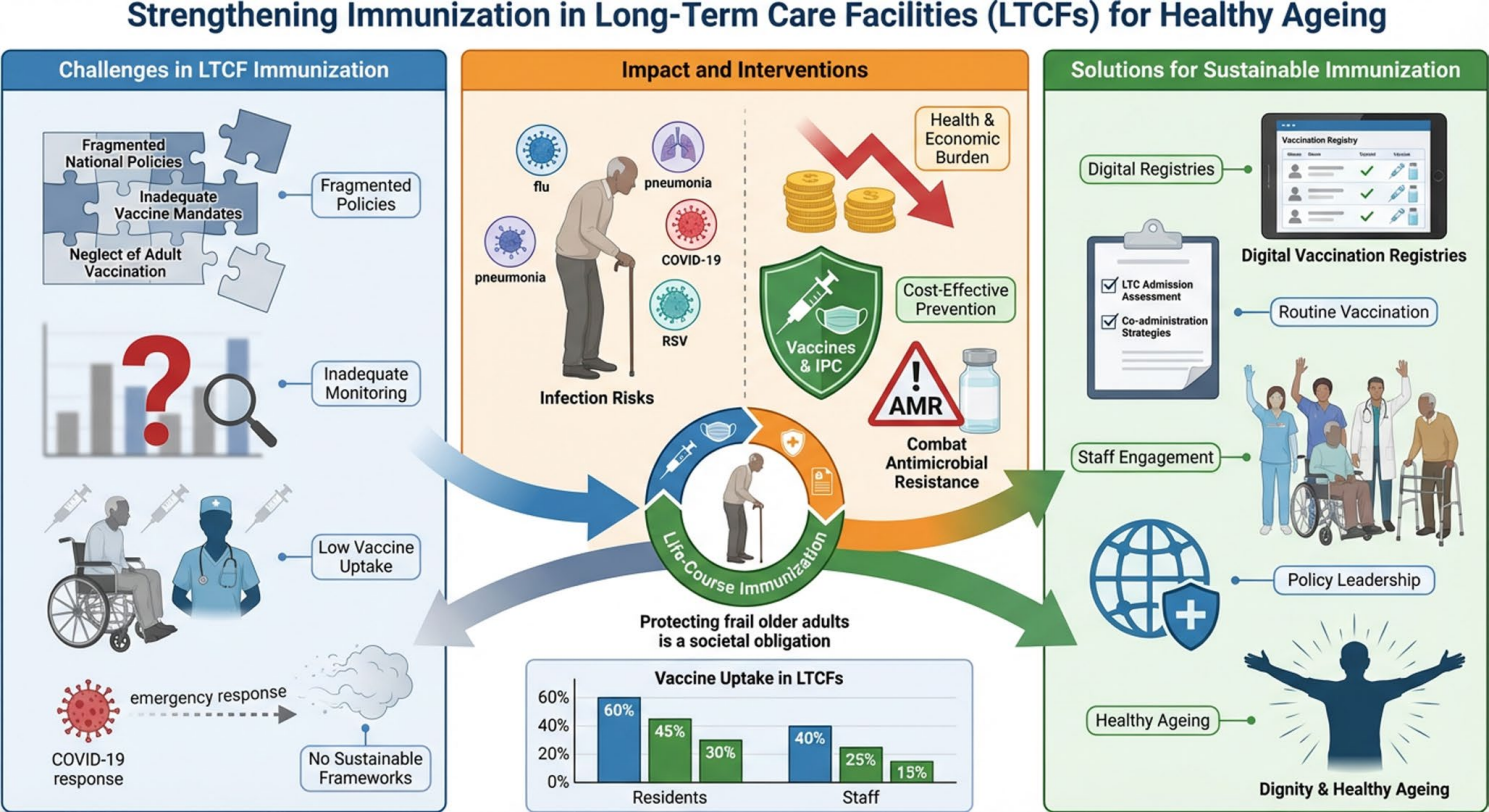

## Introduction

The global demographic landscape is undergoing a profound transformation marked by unprecedented population ageing and an escalating prevalence of non-communicable diseases (NCDs) [1]. These demographic shifts have far-reaching implications for public health systems, social care infrastructures, and the resilience of long-term care (LTC) environments. As societies experience increasing longevity, the susceptibility of older adults—particularly those with chronic health conditions—to infectious diseases (IDs) demands renewed attention and systemic response [2]. Among the most preventable threats to this population are vaccine-preventable diseases (VPDs) such as seasonal/pandemic influenza, pneumococcus, SARS-CoV-2 infection, respiratory syncytial virus (RSV), herpes zoster (HZ) and pertussis. However, despite the availability of effective vaccines, immunization coverage among older adults, usually defined as those aging 65 years or more, especially those residing in LTC facilities, remains woefully inadequate.

This disparity in immunization protection reflects a broader systemic gap: the absence of cohesive national and international frameworks to ensure routine adult vaccination across the life course. While achieving high childhood vaccination rates has been globally acknowledged as a public health priority, adult vaccination—particularly for older adults and individuals with underlying health conditions—has not received equivalent policy emphasis or funding support. This imbalance is becoming increasingly untenable as the burden of VPDs in older populations grows. Data from the World Health Organization (WHO) underscore the elevated risks associated with VPDs in people with chronic conditions: they face twice the risk of death, are 3.4 times more likely to be hospitalized, and are 1.74 times more likely to require intensive care for influenza compared to the general population [3]. 

The COVID-19 pandemic served as both a clarion call and a missed opportunity in this context. It spotlighted the vulnerability of LTC residents and older adults, prompting temporary policy innovations and emergency vaccination programs. Yet, these interventions were largely reactive and lacked permanence. As governments raced to inoculate high-risk populations against SARS-CoV-2, few established sustainable, long-term vaccination strategies for other VPDs and for COVID-19 itself. As a result, routine adult immunization—particularly within LTC settings—has reverted to pre-pandemic neglect in many jurisdictions. The pandemic, while a catalyst, has not delivered durable policy shifts in adult immunization infrastructure.

The inadequacy of adult immunization frameworks is further evidenced by a recent systematic review of 19 EU countries, which assessed the inclusion of LTC residents in National Immunization Programs (NIPs) for influenza, pneumococcal disease, and pertussis [4]. The findings revealed a fragmented landscape: not only immunization mandates were scarcely implemented due to both ethical and legal reasons, but also standardized protocols for adult vaccinations within aged care have been infrequently defined [4]. As consequence, millions of frail individuals remain unprotected, not due to vaccine hesitancy or supply limitations, but because of policy inaction. The absence of binding frameworks translates into a void of accountability—“no framework, no mandate, no protection”—and leaves a critical segment of the population at elevated risk for avoidable morbidity and mortality [5]. 

The urgency of addressing this gap is reinforced by recent developments at the global policy level. The 78th World Health Assembly (WHA), for example, adopted resolutions recognizing the importance of routine vaccination aligned with local vaccine regulations and called for equitable immunization as part of primary health care (PHC) across the life course [6]. These resolutions emphasize not only the biomedical necessity of adult vaccination but also its ethical imperative: to ensure equitable health outcomes across all ages and social strata [6]. Moreover, they advocate for the integration of immunization services into broader strategies for the prevention and control of NCDs and respiratory viruses, underscoring vaccination as a cornerstone of healthy ageing [6]. 

In parallel, WHO’s 2022 position paper on influenza vaccines provides robust epidemiological evidence supporting the prioritization of older adults and immunocompromised populations for seasonal vaccination [7]. Influenza not only poses a direct infectious threat but also acts as a catalyst for bacterial coinfections, severe cardiovascular and neurological events, including myocardial infarctions and stroke [7]. Interestingly, similar complications have been linked with other respiratory pathogens such as RSV and hMPV (human metapneumovirus), a respiratory pathogen whose vaccines are in advanced stage of development [8]. These secondary complications further burden health systems and exacerbate existing comorbidities in at-risk individuals. For example, hospitalizations due to RSV lower respiratory tract infections (LRTI) have been associated with 14% loss of independence at discharge, and 8% of patients was affected by significant loss of independence 6 months after the discharge [9]. Moreover during these last years, it became evident that vaccination such against VZV or influenza may decrease the risk of neurodegenerative disease development such as Alzheimer disease [10]. Thus, the protective benefits of vaccination extend beyond infection control—they also encompass the mitigation of systemic health deterioration and the preservation of functional independence in ageing populations.

Given these converging trends and imperatives, there is an urgent need for a paradigm shift in how vaccination is conceived, implemented, and governed within adult and LTC populations. This includes the establishment of evidence-based national immunization schedules for older adults, formal recognition of LTC residents as a priority group, and the integration of vaccination status into electronic health records and care planning processes. It also necessitates cross-sectoral collaboration among healthcare providers, policymakers, patient advocates, and international organizations to ensure that vaccination becomes a routine and expected component of adult health care, not an exception during crises.

Given this background, this paper summarized an in-person meeting held in San Servolo, Venice, Italy and promoted by the European Interdisciplinary Council on Ageing (EICA) European Platform fostering interdisciplinary analyses, high-level discussion as well as translation and dissemination of results from ageing research to various professional groups (nurses, physicians specialist in geriatrics, paediatrics, public health, general medicine, specialist in economy, and stakeholders), to policy makers, and to the general public aims to critically examine the current gaps in adult vaccination policies, with a particular focus on older adults in long-term care settings. Drawing on international data, recent policy resolutions, and public health research, we argue that routine adult immunization must be embedded into the continuum of care for ageing populations. We also explore the implications of sustained policy inaction and propose a set of actionable recommendations to strengthen immunization frameworks in alignment with global health goals. Ultimately, safeguarding the health of older adults through equitable and consistent vaccination policies is not only a medical necessity—it is a societal obligation that reflects our collective commitment to healthy and dignified ageing.

### Intrinsic factors favouring infections in long term care residents

Residents of LTC facilities represent a heterogeneous and particularly vulnerable subset of the older population, with a complex interplay of intrinsic factors that predispose them to infections. These intrinsic factors stem primarily from physiological ageing, the burden of chronic diseases, cognitive and functional decline, and compromised immune function. A central contributor may be the immunosenescence, the age-related deterioration of the immune system, which impairs both innate and adaptive immunity [11]. The changes in the immune response may be adaptive or maladaptive. When the immune response is maladaptive this results in an immunological decline which may lead to diminished ability to respond effectively to pathogens and vaccines, leading to increased susceptibility and severity of infections. It is of note that the modern vaccines tailored for older subjects have a very strong vaccine response efficiently protecting them from the VPD such influenza [12]. 

Additionally, LTC residents often experience malnutrition, which exacerbates immune dysfunction and impairs wound healing and mucosal defenses [13]. Malnutrition may be a result of dysphagia, feeding difficulties, chronic wasting diseases or inadequate dietary intake of micro- and macro-nutrients, and although it is difficult to isolate as an independent risk factor, its cumulative effect in frail individuals is significant [13]. Multiple comorbidities—such as cardiovascular disease, diabetes, chronic renal failure, cancers and chronic respiratory conditions—are highly prevalent and often accompanied by polypharmacy, further complicating the clinical picture and weakening host defenses [14]. 

Cognitive impairments are also common and can interfere with residents’ ability to maintain basic hygiene practices or to recognize and communicate symptoms of illness, thereby delaying diagnosis and treatment [15]. In many cases, infections in this population manifest atypically, with blunted febrile responses and nonspecific signs, complicating clinical recognition. Similarly, functional impairments such as immobility or incontinence increase the risk of pressure ulcers, urinary tract infections, and respiratory complications.

The use of invasive medical devices, including catheters, feeding tubes, and tracheostomies, introduces direct pathways for pathogens and is frequently associated with healthcare-associated infections [16]. Moreover, frailty, defined as a state of reduced physiological reserve and resilience, often characterizes LTC residents and correlates with poorer infection outcomes [16]. Swallowing disorders, frequent in this group, also elevate the risk of aspiration pneumonia—a leading cause of morbidity and mortality in LTC facilities [16]. 

Despite these observations, recent studies suggest that while these intrinsic factors may contribute cumulatively to infection risk, none have been consistently identified as independently predictive of infection in LTC settings [17]. Instead, the intersection of these resident-level vulnerabilities with extrinsic, facility-related conditions—such as staffing, care practices, and infrastructure—may be more determinant [17, 18]. Nonetheless, recognizing and addressing these intrinsic vulnerabilities remains vital for infection prevention strategies in LTC, especially through improved nutrition, vaccination, functional support, and tailored medical care.

### Environmental factors influencing infections in long-term care facilities

The physical and organizational environment of LTCFs plays a pivotal role in the transmission and persistence of infections. Unlike hospitals, many LTCFs lack dedicated infection prevention and control (IPC) infrastructure, making them particularly susceptible to environmental contamination [19, 20]. One of the most critical risk factors is shared infrastructure—such as multi-bed rooms, communal bathrooms, shared toilets and dining or activity areas—which promotes the rapid spread of pathogens [21]. These configurations, in combination of narrow circulation paths, limit the possibility of effective isolation, reduce zoning capabilities, and complicate outbreak containment. Similarly, another critical point is staff and visitors rotation that is harder to manage compared to hospital.

Water systems also represent a significant environmental reservoir of infection [20]. Aging plumbing, infrequent flushing & temperature control, and biofilm formation in taps and showers can harbor opportunistic pathogens like *Legionella spp.*, *Pseudomonas*, and carbapenemase-producing *Enterobacteriaceae* (CPE). Similarly, air quality and ventilation issues, especially during construction or renovation—can disseminate dust and microbial spores when appropriate controls are not in place. As well, many facilities lack mechanical ventilation, and the principle of natural ventilation is not used properly, increasing the risk of airborne transmission.

Furnishings and equipment in LTCFs frequently present cleaning challenges [19]. Soft furnishings are difficult to disinfect, and shared medical equipment is often inadequately sanitized, particularly non-critical devices. There is also limited evaluation and standardization of cleaning methods. Environmental surfaces, especially high-touch areas in residents’ rooms and shared spaces, are frequently contaminated with clinically significant pathogens [22]. Inconsistent cleaning protocols & cleaning responsibilities, insufficient staff training, and inadequate availability of broad-spectrum disinfectants exacerbate this risk.

Laundry, bedding, and waste management are often overlooked aspects of IPC [22]. Improper handling of soiled linens can lead to aerosolization of infectious agents, while poor waste segregation and disposal increase the risk of environmental contamination. These factors are compounded by the widespread lack of IPC-trained personnel, limited surveillance, inadequate policy frameworks, and role ambiguity in many LTCFs.

In summary, environmental conditions within LTCFs are central to the infection burden experienced by residents. Tailored guidelines, investment in infrastructure, dedicated IPC teams, leadership, regular environmental audits, and staff education are essential to mitigate these risks and improve infection outcomes in these vulnerable settings.

### Economic burden of infectious diseases in long-term care facilities

IDs in LTCFs impose a substantial yet frequently underrecognized economic burden on healthcare systems [23]. LTC residents—characterized by advanced age, frailty, multimorbidity, and immunosenescence—are particularly vulnerable to infections due to factors such as shared living spaces, close contact care, and limited infection prevention infrastructure. As a result, healthcare-associated infections (HAIs) are widespread in LTC settings. A recent European study spanning nine countries found that over 57% of residents experienced at least one HAI within a year, with respiratory and urinary tract infections being most prevalent [24]. Notably, 4.3% of these infections required hospitalization, and 4.5% were associated with death, particularly from respiratory causes [24]. 

The economic costs associated with these infections are multifaceted [25]. They include direct medical expenses—such as hospital transfers, diagnostics, and antimicrobial therapies—as well as indirect costs stemming from staff time, infection management efforts, prolonged disability, and decreased quality of life. In U.S. nursing homes alone, hospitalizations due to HAIs account for an estimated \$637 million to \$2 billion annually, with additional costs of \$38–137 million for antimicrobial therapy [25]. However, in Europe and other regions, there remains a scarcity of comprehensive economic studies, especially in terms of long-term consequences like hospital readmissions and persistent disability.

The burden of infection extends beyond finances, measured in non-monetary units such as disability-adjusted life years (DALYs), encompassing both years of life lost (YLL) due to premature death and years lived with disability (YLD). These outcomes highlight the societal impact of infections in LTCFs and the pressing need for economically sound prevention strategies.

Preventive interventions, particularly vaccination and robust infection control practices, offer a promising pathway to mitigate both health and economic losses. Studies on influenza and COVID-19 vaccination in older populations indicate strong cost-effectiveness, especially when evaluated from a societal perspective [26]. Moreover, clinical best practices—such as rigorous hand hygiene, environmental sanitation, and early screening—have demonstrated favorable economic profiles. While direct cost-effectiveness analyses within LTCFs are limited, particularly for COVID-19 infection [27], extrapolation from broader elderly population data suggests that prioritizing infection prevention in LTC environments not only improves outcomes but also represents a financially sustainable policy approach [27]. 

Ultimately, incorporating economic assessments into LTC planning and infection prevention policy development is essential [28]. Doing so ensures that resources are allocated effectively and that preventive strategies are recognized not just as clinical necessities but as critical investments in the sustainability of long-term care systems.

## Burden of infectious respiratory diseases in long-term care facilities

Respiratory tract infections (RTIs), particularly pneumonia, represent a significant burden in LTCFs, second only to urinary tract infections in prevalence [29]. Epidemiological data underscore the disproportionate impact on LTC residents, with studies reporting incidence rates of pneumonia ranging up to 28% and associated hospitalization and mortality rates far exceeding those in community-dwelling older adults [29, 30]. Common pathogens include influenza viruses, RSV and pneumococcus, with many outbreaks linked to vaccine-preventable strains, yet vaccination policies at the point of LTCF entry remain inconsistent across countries [29]. 

The functional consequences of RTIs are profound. Acute infections often precipitate distress and declines in activities of daily living (ADL), particularly in residents with dementia, who may exhibit atypical symptoms and experience heightened discomfort [31, 32]. Studies have shown that pneumonia leads to a marked increase in care needs and a decline in vitality and psychological well-being—particularly following hospitalization. The INCUR and FINE studies revealed that pneumonia episodes are associated with worsened intrinsic capacity and prolonged ADL recovery, even in survivors [31, 32]. In addition, the quality of life tends to decline, especially in those requiring hospital-level care.

For LTC staff and facilities, the burden includes increased surveillance, care demands, and the psychological toll of managing outbreaks [33, 34]. Staff often face challenges in early recognition and response, complicated by time pressures, lack of training, and high resident-to-caregiver ratios. Facilities must coordinate infection prevention, manage isolation protocols, and often deal with downstream consequences such as antimicrobial resistance and litigation risks.

In summary, RTIs in LTCFs not only result in high morbidity and mortality but also lead to significant and often prolonged functional decline in residents. Addressing this burden requires integrated strategies that include preventive vaccination, staff training, early recognition protocols, and long-term functional support to mitigate lasting consequences on residents’ independence and well-being.

### An exemplified Story, not to forget: COVID-19 in Long-Term care institutions

The COVID-19 pandemic offered a stark and painful lesson about the fragility of long-term care institutions [35]. Nursing homes became epicentres of infection and mortality, driven by the perfect storm of close quarters, vulnerable residents, and often asymptomatic viral transmission by staff and visitors. Despite widespread vaccine rollouts and declining public attention, surveillance data from the U.S. revealed that outbreaks have remained a persistent background threat in these facilities, with infection still leading to hospitalization in approximately 10% of cases [36]. Studies showed that rapid point-of-care testing, especially when implemented with real-time results and facility-level autonomy, significantly improved outbreak detection and responsiveness—highlighting the importance of timely diagnostics and surveillance [37]. However, the discontinuation of routine testing and underreporting, especially after policy changes, have only obscured the ongoing burden. Moreover, vaccination campaigns—including mid-season boosters—have proven effective, though uptake among residents and staff remains a challenge [38]. This story, rooted in lived experience and reinforced by data, reminds us that complacency in these settings is dangerous. Sustained vigilance, robust testing, staff immunization, and infection control practices are essential to protect those in our care who are least able to protect themselves.

## Healthcare associated infections (hai) in Ltc residents

The fourth wave of the Healthcare-Associated Infections and Antimicrobial Use in Long-Term Care Facilities (HALT-4) project, coordinated by the European Centre for Disease Prevention and Control (ECDC), provides critical insights into the evolving infection landscape across European LTCFs [24]. Conducted between 2023 and 2024 across 18 EU/EEA countries, HALT-4 employed both point prevalence surveys (PPS) and a year-long longitudinal study to assess the burden of HAIs and patterns of antimicrobial use in general nursing homes, residential homes, and mixed LTCFs [24]. The point prevalence survey revealed an HAI prevalence of 3.1%, with urinary and respiratory tract infections accounting for the majority of cases [24]. Meanwhile, the antimicrobial use prevalence stood at 4.1%, showing a slight decline from previous HALT rounds. Worryingly, 21.3% of antimicrobial prescriptions lacked an end or review date, raising concerns about prolonged or inappropriate usage [24]. 

The longitudinal cohort study deepened these findings by documenting that nearly half of LTCF residents experienced at least one HAI over a 12-month period, with respiratory and urinary infections dominating [24]. These infections were not only frequent but also carried severe outcomes, including hospitalizations and deaths—especially among older, frail residents with multiple comorbidities. The study also highlighted a significant gap in infection prevention and control (IPC) infrastructure and antimicrobial stewardship (AMS): less than one-quarter of LTCFs had surveillance programs for antimicrobial consumption, and many lacked even basic AMS elements.

HALT-4 underscores the urgent need for systemic improvements in infection surveillance, staff training, and antimicrobial prescribing practices within LTCFs [24]. Key recommendations include strengthening IPC competencies, ensuring availability of microbiological testing, and implementing targeted AMS programs, particularly for urinary tract prophylaxis and diagnostic-guided treatments. With standardized data now available across Europe, HALT-4 provides an evidence base for shaping future interventions and policies to mitigate HAIs and promote responsible antimicrobial use in vulnerable LTC populations.

### Non-Pharmacological infection prevention and control measures

In LTCFs, non-pharmacological IPC measures are essential components of a comprehensive strategy to reduce the risk of HAIs, especially given the vulnerability of residents due to age, multimorbidity, and functional impairment. These measures are grounded in a clear understanding of pathogen characteristics, modes of transmission (contact, droplet, airborne), and the resident environment. At the core of these practices, other than vaccinations, are other standard precautions, which should be applied by all health-care workers, at all times, for all residents, and in all care settings. When applied consistently, standard precautions can prevent the transmission of microorganisms between residents, health workers and the environment [39]. The main key elements of standard precautions include rigorous hand hygiene, effective environmental cleaning, respiratory hygiene, appropriate use of personal protective equipment (PPE), and precautions during invasive procedures.

Hand hygiene, recognized as the cornerstone of IPC, is most effective when alcohol-based hand rub (ABHR) is used at the five moments recommended by the World Health Organization: before patient contact, before an aseptic procedure, after exposure to body fluids, after patient contact, and after contact with the patient’s environment [40, 41]. Despite its proven efficacy, compliance in LTCFs remains low, averaging 31%, with consumption rates below optimal levels [42]. A multimodal strategy—encompassing system change, training, feedback, and reminders—is recommended to improve adherence. Similarly, environmental cleaning must be systematic, targeting high-touch surfaces and shared medical equipment, with frequency and methods tailored based on risk assessments and pathogen persistence [43]. 

Respiratory hygiene—especially during seasonal outbreaks—includes mask use by symptomatic residents and staff, respiratory etiquette (e.g., covering mouth/nose during coughing), and the use of FFP2 masks during aerosol-generating procedures [44]. Ventilation and facility design also play critical roles; adequate air exchange and separation of clean and dirty circuits help reduce airborne contamination. It is common that current LTCs have rooms and bathrooms in common between two or more residents: architectural features such as single rooms with private bathrooms and clearly defined utility areas further limit cross-contamination [45]. Similarly, shared medical instruments in common might be another important source of infections in LTCs [46]. 

Additionally, preventing infections associated with invasive procedures—like urinary catheterization or vascular access—requires careful documentation, daily reassessment, and timely removal of devices [47]. Altogether, these non-pharmacological interventions form a robust, cost-effective defence against infections in LTCFs. However, their success depends on staff training, institutional support & leadership, and consistent application across all levels of care.

### Prevention of antimicrobial resistances in adults and older adults

According to the estimates from Global Burden of Disease Group, each year, an estimated 7.7 million deaths are associated with bacterial infections [48], and 4.95 million deaths are associated with antimicrobial resistance, with 1.27 million deaths due to bacterial pathogens exhibiting resistance to available antibiotics. Moreover, available projections – mostly due to high income countries, suggest that AMR to 3rd line antibiotics could increase up to 2.1 times in 2035 compared to current estimates, with a similarly increased economic and social burden [48]. In other words, AMR in adults and older adults represents a critical public health priority, given their heightened vulnerability due to factors such as chronic conditions, polypharmacy, malnutrition, and increased exposure to healthcare environments like hospitals and nursing homes [49]. In this population, infections are more frequent and severe, often resulting in higher mortality. Unfortunately, as recently stressed [48], investments in AMR research and development lag behind other conditions with smaller health burden. Moreover, availability of novel antimicrobial drugs not only lag well behind the increasing burden of AMR in real-world settings, but their cost per treatment course may limit their availability in high-income settings, ultimately increasing the usage of older and more affordable drugs in middle- and low-income settings, but also the risk for increased AMR [50, 51]. Key strategies to curb AMR therefore include robust IPC measures, improved sanitation and hygiene (WASH), and the promotion of appropriate antibiotic use through antimicrobial stewardship programs [49]. Additionally, expanding and guaranteeing access to affordable and effective vaccines—particularly those targeting high-priority pathogens like *Streptococcus pneumoniae*, *Escherichia coli*, and *Mycobacterium tuberculosis*—could significantly reduce the need for antibiotics and prevent resistant infections [52]. Investment in surveillance, diagnostics, public education, and equitable access to essential health services further strengthens the response. Without urgent and coordinated action, including fostering innovation and ensuring global access to new antimicrobials and vaccines, the burden of AMR in older populations will continue to escalate.

### Prevention of Anti-Microbial resistances in children: focus on a Life-Long approach

When considering older people living in LTCs, it is important to remember that preventing AMR is a life-long approach. Neonates and infants are particularly vulnerable due to their immature immune systems and the necessity for empirical antibiotic use in critical care settings [53]. However, this early exposure—often excessive and prolonged—disrupts the microbiota and contributes to long-term consequences such as dysbiosis, weakened immune development, increased risk of chronic conditions like asthma and obesity, and the colonization of multi-drug-resistant organisms. These effects can ripple across generations, with evidence suggesting a bi-directional transmission of resistant pathogens between children and their older caregivers. Implementing antimicrobial stewardship from the neonatal period is vital, including strategies to minimize unnecessary antibiotic use, optimize treatment duration, and adopt effective preventive measures such as targeted prophylaxis (e.g., fluconazole for invasive fungal infections). Such efforts not only protect children’s immediate health but also shape a healthier microbial and immunological trajectory throughout life, helping to curb the global spread of AMR.

### Vaccine uptake rates in residents in Long-Term care facilities

It is widely known that appropriate vaccination can contribute to reducing AMR burden [54]. Despite being among the most vulnerable to infectious diseases, vaccine uptake rates among residents of LTCFs remain consistently suboptimal across many countries [55]. During the COVID-19 pandemic, LTCF residents experienced disproportionate mortality—up to 30–60% of deaths in the first wave in Europe—yet recognition of this population in national immunization policies has been limited [56–58]. In Europe, robust LTCF-specific vaccine coverage data are scarce; the HALT-4 point-prevalence survey (2023–2024), based on structured facility reporting, indicated high COVID-19 and influenza vaccination coverage among residents, but with very wide variation between countries (~ 15% to nearly 100%). These figures should be interpreted cautiously, as definitions of ‘fully vaccinated’ differed and comparable data are otherwise limited [59]. 

In the United States, influenza vaccine coverage stabilized around 61–62%, COVID-19 booster uptake declined slowly after peaking near 50%, and RSV vaccination remained low, reaching only around 25% by mid-2025 [60]. These patterns reflect systemic challenges, including limited LTCF-specific policy mandates, inconsistent monitoring, logistical barriers, low onsite medical coverage, and difficulties tracking vaccination status in residents admitted continuously [60]. 

Compounding the issue are socio-behavioral barriers—such as vaccine hesitancy, misinformation, ageism, and low risk perception among residents, families, and staff—as well as operational constraints like staff shortages and high turnover [61]. Most existing strategies focus broadly on older adults without tailoring implementation to the LTCF setting. As a result, coverage for other recommended vaccines—including pneumococcal, herpes zoster, and Tdap—remains poorly documented and largely invisible, despite being included in national schedules [61]. 

To improve protection for LTCF residents, a shift is needed toward structured, on-site, life-course vaccination approaches. These should include digital tracking of doses, routine assessment at admission, co-administration strategies, seasonal campaigns, and stronger inclusion of LTCFs in national immunization plans. Without systematic efforts to monitor and act on coverage gaps, the goal of equitable vaccine protection in LTCFs will remain out of reach.

### Vaccine uptake rates among staff working in Long-Term care facilities

Despite the well-documented benefits of vaccination in protecting both staff and vulnerable residents in LTCFs, vaccine uptake among healthcare workers remains alarmingly low [61]. This issue is particularly evident with seasonal influenza vaccination, which is still met with scepticism and resistance among nursing staff, despite national recommendations and professional endorsements. Yet studies consistently demonstrate that higher staff vaccination rates for respiratory pathogens such as influenza and pneumococcus could significantly reduce the incidence of outbreaks and mortality among residents—by as much as 20–40% [62]. Interestingly enough, inappropriate vaccination rates among healthcare workers have been documented also for other immunizations whose efficacy has been otherwise extensively proven. For instance, while hepatitis B vaccination rates exceed 90% among Swiss nursing staff, flu vaccination coverage hovers around 25%, primarily due to doubts about vaccine effectiveness [63], and similarly inappropriate vaccination rates have been documented also for VZV [64], a case where avoiding natural infection among the staff could radically reduce the risk for outbreaks among residents of LTCFs. Organizationally, LTCFs may implement mandatory vaccination policies, but this enforcement may be limited by underlying blueprint of legal framework which in most European settings may be limited to occupational consequences, such as job termination from the employer implementing the mandate, rather than physical compulsion. Consequently, the employee refusing recommended or even mandated vaccine(s) may ultimately find another employer with a more “flexible” approach towards vaccination requirement. Structured approaches like infection control checks, regular titer assessments, and “train-the-trainer” strategies can support higher uptake. Ultimately, shifting from passive nudging to proactive education and empowerment (form >inform >perform) is essential to building trust and improving vaccination rates among LTCF personnel.

## Available vaccines for preventing infections in residents in long-term care facilities

### COVID-19

The COVID-19 pandemic exposed the acute vulnerability of LTCF residents, whose advanced age, frailty, and multiple comorbidities placed them at disproportionately high risk of severe outcomes [65, 66]. During the early waves of the pandemic, LTCFs accounted for a significant proportion of COVID-19 mortality, with peak deaths occurring in the first and second waves [67]. Vaccine rollout in LTCFs, particularly in the UK, provided substantial protection, with early studies demonstrating strong effectiveness in reducing hospitalization and mortality. However, the benefits were challenged by several factors: the rapid waning of immunity, especially in older and frail adults; the limited durability of booster doses; and the modest protection against mild-to-moderate disease during subsequent variant waves such as Omicron [68]. 

Evidence suggests that COVID-19 infection itself may accelerate immunosenescence—marked by diminished naïve T-cell populations and increased immune exhaustion—further complicating vaccine responsiveness in older adults [69]. While hybrid immunity (from infection and vaccination) offered enhanced short-term protection, studies confirmed that this, too, diminished over time [70]. Among healthy adults, protection from a second booster declined from 24% effectiveness in the first two months to just 1.7% after six months [71]. Given these trends, LTCF residents—already experiencing a baseline of immune decline—may require more frequent or alternative boosting strategies to sustain protection.

Despite widespread vaccine access and the initial success of rollouts, persistent logistical and immunological challenges have highlighted the need for more tailored approaches. Future directions should focus on optimizing booster schedules for frail populations, integrating immunological monitoring into LTCF protocols, and investing in next-generation vaccines that offer longer-lasting and transmission-blocking protection. As COVID-19 becomes endemic and linked to seasonal influenza in terms of recurring threats, LTCFs must remain a central focus in immunization policy and preparedness planning.

Since 2023, vaccine manufacturers and regulators have shifted to variant-adapted formulations (e.g., XBB-adapted monovalent vaccines); cohort and surveillance studies reported moderate short-term reductions in COVID-19-related hospitalisation among older adults following 2023–2024 updated boosters, and the EMA has recommended seasonal updating of authorized vaccine antigen composition to match circulating variants [72]. 

### Respiratory vaccines in Long-Term care: Influenza, Pneumococcal, and RSV

Respiratory tract infections (RTIs) remain the leading cause of morbidity and mortality among residents of long-term care facilities (LTCFs), representing not only a direct threat to individual health but also a major challenge for institutional resilience and healthcare systems at large. Among these infections, influenza [73], pneumococcal disease, and respiratory syncytial virus (RSV) are responsible for the majority of outbreaks and hospitalization episodes, with significant consequences for functional decline, quality of life, and mortality. The overlapping seasonality and clinical presentation of these pathogens further complicate early diagnosis and outbreak control, often leading to delayed intervention and inappropriate antibiotic use. Vaccines targeting these respiratory pathogens therefore serve as complementary and synergistic tools that can reduce both the health and economic burden associated with respiratory infections in frail populations, such as in the case of influenza high dose or adjuvanted forms.

Older adults in LTCFs typically present with a cluster of vulnerabilities—including immunosenescence, multimorbidity, malnutrition, cognitive impairment, and functional dependence—that collectively weaken immune defense mechanisms and increase the risk of infection and severe outcomes. Immunosenescence, in particular, diminishes the ability to mount a robust immune response to natural infection and vaccination, underscoring the importance of using vaccine formulations specifically optimized for older adults. Recent evidence highlights that high-dose and adjuvanted influenza vaccines can elicit stronger humoral and cellular responses, leading to meaningful reductions in influenza-related hospitalizations and deaths [74, 75]. Similarly, newer pneumococcal conjugate vaccines, such as PCV20 and PCV21, extend protection against a broader range of circulating serotypes and show improved immunogenicity compared with earlier formulations [76–79]. The recent licensure of RSV vaccines for older adults represents a major milestone in respiratory disease prevention, offering an additional layer of protection in a population that experiences disproportionately high RSV-related morbidity and mortality [80–83]. Randomized trial efficacy and subsequent national programme evaluations (e.g., Scotland’s RSVpreF rollout) show RSV vaccine effectiveness against RSV-related emergency department visits and hospital admissions in older adults commonly in the 60–80% range; early national data indicate sizable reductions in hospitalisations among vaccine-eligible older adults after programme introduction [84]. Safety profiles are generally favorable, though rare events such as Guillain-Barré syndrome have been observed in limited cases (particularly related to recombinant vaccines), underscoring the importance of continuous monitoring [85]. 

Despite the availability of these safe and effective vaccines, uptake remains inconsistent and insufficient. This shortfall stems from fragmented immunization policies across countries, lack of standardized protocols for adult vaccination within LTCFs, logistical barriers to vaccine delivery, and persistent misinformation or vaccine hesitancy among both residents and staff. Furthermore, vaccination data in LTCFs are often incomplete or poorly integrated into electronic health records, hampering systematic monitoring and performance assessment [86]. Implementing integrated immunization approaches—such as co-administration of vaccines, digital tracking systems, and mandatory vaccination assessments at the time of LTCF admission—could substantially improve coverage, reduce missed opportunities, and streamline coordination between healthcare providers.

To optimize protection, a coordinated, multidisciplinary effort is required, engaging physicians, nurses, infection control teams, public health authorities, and LTCF administrators. Education and advocacy campaigns should emphasize the dual benefits of vaccination: protecting residents and reducing staff absenteeism during outbreak seasons. Addressing staff immunization is particularly critical, as healthcare workers serve as both caregivers and potential vectors of infection; yet, their vaccination rates often remain below 30% for key respiratory vaccines. A cultural shift toward perceiving staff vaccination as a professional responsibility, supported by institutional policies and national mandates, is essential.

Ultimately, respiratory vaccination in LTCFs should not be treated as an episodic or seasonal measure but as a structural, continuous component of comprehensive infection prevention and healthy ageing strategies. By embedding vaccination within routine care planning, leveraging digital infrastructure, and fostering a culture of prevention, LTCFs can strengthen resilience against respiratory threats while safeguarding the dignity, autonomy, and quality of life of their residents.

### Pertussis

Despite long-standing perceptions that pertussis (whooping cough) is a childhood disease, recent data show a rising incidence among older adults, particularly those aged 65 and above, many of whom reside in LTCFs. In this population, waning immunity—typically occurring 4 to 12 years after initial vaccination—combined with the presence of chronic conditions such as COPD, asthma, or obesity, increases susceptibility to severe pertussis-related complications [87]. Clinical recognition is often delayed, as pertussis in adults mimics bronchitis, influenza, or the common cold, with a protracted cough that is frequently misattributed to pre-existing comorbidities [88]. These delays not only impair timely diagnosis and treatment but also heighten the risk of transmission, especially during the early phase of infection when individuals are most contagious but least likely to be accurately diagnosed.

In LTCFs, pertussis-related clusters may be underreported, but chronic cough among residents—and particularly among under-vaccinated healthcare workers—raises concerns about silent transmission [89]. Staff members, often unaware of their susceptibility or the potential to infect vulnerable residents, are rarely vaccinated despite being key vectors. Moreover, adult pertussis surveillance remains passive, missing many mild or atypical cases [89]. This situation is compounded by low public and professional awareness, with many healthcare providers failing to recognize the relevance of pertussis vaccination in the elderly or in caregivers such as grandparents [89]. 

Given these gaps, pertussis vaccination in nursing homes should be reconsidered as a priority. The inclusion of pertussis boosters (e.g., Tdap) into adult and staff immunization protocols, supported by electronic vaccination registries and personalized outreach, could significantly reduce the burden of disease. Improved coverage would not only protect frail residents but also reduce absenteeism and transmission risk among healthcare personnel, aligning with broader goals of healthy ageing and infection prevention in institutional care settings. However, it should be recognized that for pertussis, the literature available in older people is still limited.

### Herpes Zoster

Shingles, or herpes zoster (HZ), is a painful and often debilitating condition caused by the reactivation of latent varicella zoster virus (VZV), with its incidence and severity increasing markedly with age and immune senescence [90]. Residents of nursing homes are particularly vulnerable due to advanced age, immunosuppression, and underlying comorbidities. In England and Wales, adults aged 70–79 face an annual incidence of 790–880 cases per 100,000, and the condition carries a lifetime risk of 1 in 4 [91]. Complications such as post-herpetic neuralgia (PHN)—a chronic pain syndrome—occur in up to 25% of cases in older adults, while more severe outcomes, including hospitalisation, ophthalmic involvement, and even death, are more likely in this population [92]. 

The introduction of the shingles vaccination programme in the UK, initially using the live-attenuated vaccine (LZV), resulted in notable public health gains, including 40,500 fewer GP consultations and nearly 2,000 fewer hospitalisations over five years [93]. The coverage of HZ vaccination is still low in the UK, even if a linear increase by age group was reported [94]. More recently, the recombinant, adjuvanted vaccine (RZV) has been adopted as the preferred vaccine for individuals over 50, and for immunocompromised populations with 18 years and more [95]. Clinical trials have shown that adjuvanted vaccine to be over 97% effective in preventing shingles and 76% effective against PHN in adults aged 65 and above, with sustained protection [96]. 

Shingles vaccination has further demonstrated broader health benefits. Large cohort studies suggest associations between herpes zoster vaccination and reductions in cardiovascular events and dementia diagnoses [97], underscoring the systemic impact of preventing VZV reactivation. Vaccination against HZ is suitable for co-administration with influenza, pneumococcal, RSV, and COVID-19 vaccines, offering a practical strategy for integrated immunisation in nursing home settings.

Despite these advantages, vaccine uptake remains variable, constrained by limited awareness, logistical challenges, and inconsistent inclusion in long-term care immunisation protocols. Strengthening uptake in nursing homes through structured vaccination campaigns, electronic registries, and routine vaccination at admission could significantly reduce disease burden, enhance resident well-being, and prevent avoidable healthcare utilisation.

### Discussion: facing the challenge in Ids prevention, control and treatment in Ltc residents

The roundtable discussion highlighted the multifaceted and persistent challenges in preventing, controlling, and treating IDs in LTC settings. Experts emphasized that LTC residents represent a unique and highly vulnerable population due to advanced age, immunosenescence, frailty, and a high burden of chronic diseases [98]. These intrinsic factors are compounded by environmental and organizational vulnerabilities, including shared spaces, inadequate infection control infrastructure, staff turnover, and limited access to on-site IPC professionals.

One of the key points raised was the fragmented nature of adult immunization policies across countries. Participants stressed that although vaccines exist for many high-risk infections—such as influenza, pneumococcal disease, pertussis, COVID-19, RSV, and herpes zoster—coverage remains suboptimal in LTCFs due to lack of mandates, poor data tracking, and weak integration into care planning. The discussion also pointed to the missed opportunity during the COVID-19 pandemic to institutionalize vaccination programs as a standard of care for LTC residents and staff.

Participants underscored the importance of staff immunization, noting that low uptake among healthcare workers poses a significant barrier to resident protection. There was consensus on the need for robust education, behavioural interventions, and role modelling to shift perceptions and increase vaccine acceptance among caregivers. Additionally, the panel called for systemic improvements, including the establishment of adult immunization registries, routine vaccination assessments at admission, and the use of digital tools to monitor and prompt vaccine delivery.

Beyond vaccination, the discussion addressed antimicrobial stewardship, diagnostic capacity, and infection surveillance. It was acknowledged that many LTCFs lack microbiological support, making targeted treatment difficult and fostering overuse of broad-spectrum antibiotics. The need for tailored IPC (infection prevention and control) protocols & teams, leadership in IPC, regular staff training, and investment in infrastructure—such as ventilation, sanitation, and single-occupancy rooms—was strongly advocated.

Finally, the participants highlighted the role of nursing clinical leadership. Given that nurse leaders are closely involved in daily care processes, vaccination programs, and infection prevention practices, their contribution is essential in bridging organizational policies and bedside implementation. It might be valuable to highlight their role in staff education, mentoring, and fostering a culture of safety, perhaps in the section following staff immunization. In this sense, the importance of training and continuous education for nurses, as their knowledge and skills are fundamental to implementing sustainable infection prevention and control practices in LTCFs.

While this consensus provides a comprehensive overview of infectious disease prevention and vaccination strategies in long-term care facilities, several limitations should be acknowledged. First, the recommendations reflect expert opinion based on available evidence up to early 2025; data gaps remain regarding the long-term effectiveness and cost-effectiveness of newer vaccines such as RSV and higher-valent pneumococcal formulations in frail populations. Second, heterogeneity in national health systems, LTCF structures, and regulatory frameworks limits the generalizability of specific policy suggestions across Europe. Third, few randomized or longitudinal studies directly compare vaccination strategies or infection control interventions within LTCFs, necessitating further empirical validation. Finally, while this document represents multidisciplinary consensus, input from patient representatives and frontline care staff was limited, underscoring the need for more participatory approaches in future consensus processes.

### Concluding remarks and practical implementation steps

The consensus of the European Interdisciplinary Council on Ageing underscores that preventing infectious diseases in long-term care facilities requires not only scientific evidence but also practical, system-level implementation. Translating these recommendations into action demands a coordinated approach that bridges policy, clinical practice, and education. In practical terms, facilities should adopt structured immunization programs that include systematic vaccination assessments upon admission, integration of digital registries to monitor coverage, and promotion of co-administration strategies to simplify logistics. Strengthening staff vaccination policies, supported by clear occupational guidelines and continuous education, is equally essential to protecting both caregivers and residents.

At the organizational level, infection prevention and control (IPC) teams should be embedded as permanent structures within LTCFs, ensuring sustained leadership, training, and accountability. Close collaboration between public health authorities and LTC administrators can align vaccination initiatives with broader healthy ageing and antimicrobial resistance (AMR) strategies. Finally, sustained investment in infrastructure, surveillance systems, and research on vaccine effectiveness in frail populations will be critical to closing current evidence gaps. Implementing these steps will help transform vaccination and infection control from isolated interventions into integral components of quality care—strengthening resilience, safeguarding dignity, and advancing the goal of healthy ageing across Europe.

## Data Availability

No datasets were generated or analysed during the current study.
